# P-2112. Assessing Incidence, Risk Factors, and Outcomes of BK Virus-Related Complications Following Kidney Transplantation

**DOI:** 10.1093/ofid/ofaf695.2276

**Published:** 2026-01-11

**Authors:** Bin Ni, Scott Sanoff, Xunrong Luo, J Eric Jelovsek, Barbara D Alexander

**Affiliations:** Duke University Medical Center, Durham, NC; Duke University Medical Center, Durham, NC; Duke University Medical Center, Durham, NC; Duke University, Durham, North Carolina; Duke University School of Medicine, Durham, North Carolina

## Abstract

**Background:**

BK polyomavirus reactivation after kidney transplant can cause complications like BK virus-associated nephropathy (BKVAN) and ureteral stenosis. Rates vary by transplant practices and immunosuppression. This study assessed the epidemiology, risk factors, and outcomes of BK reactivation in kidney transplant recipients.

**Table 1:**
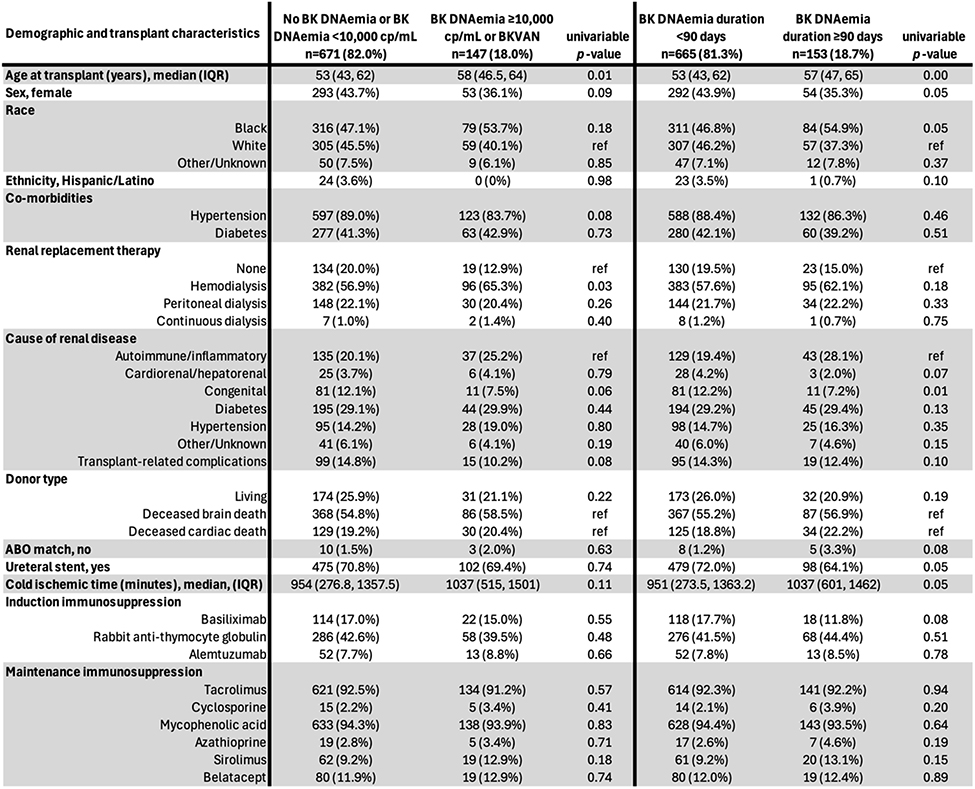
Demographics and transplant characteristics of renal transplant recipients with and without high-level BK DNAemia, and with and without persistent BK DNAemia.

**Figure 1:**
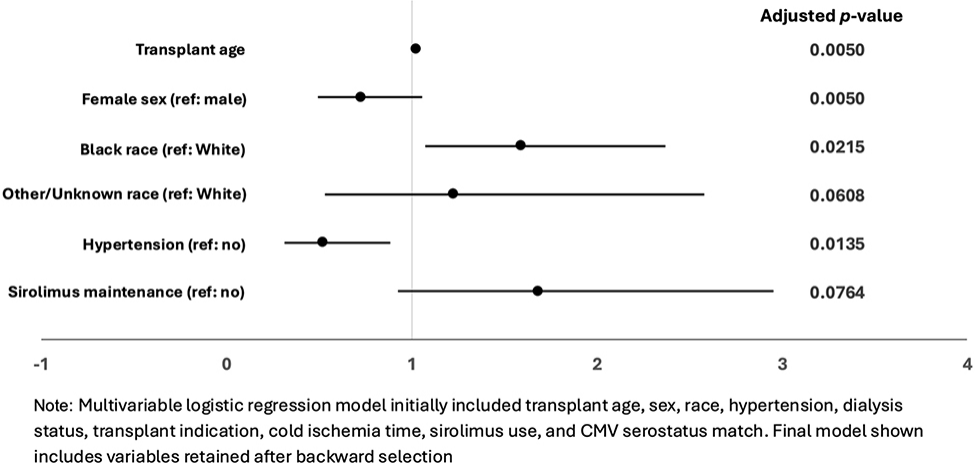
Factors associated with high-level BK DNAemia (≥10,000 copies/mL) or BK nephropathy after kidney transplant.

**Methods:**

We conducted a retrospective study of kidney transplants at Duke University Hospital (2016–2020), comparing recipients with and without high-level (≥10,000 copies/mL) and persistent ( >90 days) BK DNAemia. Logistic regression was used for analysis; variables with *p*-value < 0.20 in univariable analyses were included in the multivariable model.

**Figure 2:**
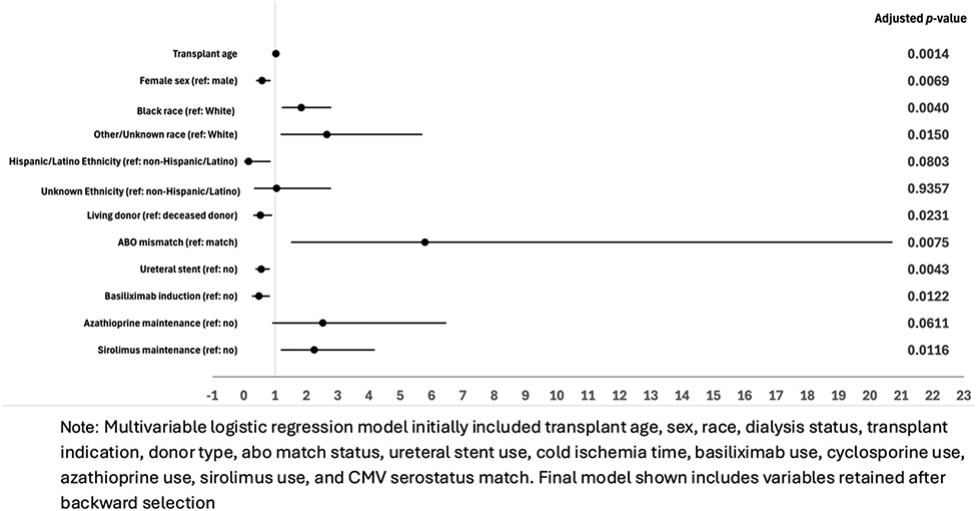
Factors associated with persistent BK DNAemia (>90 Days) after kidney transplant

**Results:**

Among 818 recipients with ≥30 days of follow-up, 301 (36.8%) developed detectable BK DNAemia, including 147 (18%) with high-level DNAemia, 153 (18.7%) with persistent DNAemia, and 47 (5.7%) with proven, presumptive, or probable BKVAN. Ureteral stenosis/obstruction occurred in 37 (4.5%) patients. BKVAN was linked to higher peak viral load (median 210,000 vs. 3,095 copies/mL; *p*< 0.001) and longer viremia (median 398 vs. 77.5 days; *p*< 0.001). Older age (aOR 1.02, 95% CI 1.01-1.04) and Black race (aOR 1.59, 95% CI 1.07-2.37) were associated with high-level BK DNAemia, while female sex (aOR 0.73, 95% CI 0.49-1.06) and hypertension (aOR 0.52, 95% CI (0.31-0.89) were associated with lower odds. Persistent BK DNAemia was more likely with older age (aOR 1.03, 95% CI 1.01-1.04), Black/Other race (aOR 1.84/2.65, 95% CI 1.22-2.79/1.17-5.71), ABO mismatch (aOR 5.78, 95% CI 1.51-20.72), and any sirolimus use (aOR 2.25, 95% CI 1.18-4.18), but less likely with female sex (aOR 0.58, 95% CI 0.39-0.86), living donors (aOR 0.53, 95% CI 0.30-0.90), and ureteral stent (aOR 0.55, 95% CI 0.37-0.83). Those with high-level BK DNAemia had higher odds of biopsy (OR 1.68, 95% CI 1.16-2.43) and rejection (OR 1.85, 95% CI 1.19-2.83). Those with persistent BK DNAemia had lower odds of return to dialysis at 2y (OR 0.11, 95% CI 0.006-0.49).

**Conclusion:**

High-level and persistent BK DNAemia are influenced by various factors. While high-level BK DNAemia increases biopsy and rejection rates, persistent BK DNAemia correlates with lower risk of return to dialysis at 2y, suggesting the need for further investigation into its clinical implications.

**Disclosures:**

Xunrong Luo, MD, PhD, Diabetes-Free, Inc: Use of ECDI-fixed cell tolerance J. Eric Jelovsek, MD, MMEd, MSDS, Collamedix: Board Member

